# Immunogenic cell death-led discovery of COVID-19 biomarkers and inflammatory infiltrates

**DOI:** 10.3389/fmicb.2023.1191004

**Published:** 2023-05-09

**Authors:** Jianzhen Zhuo, Ke Wang, Zijun Shi, Chunlei Yuan

**Affiliations:** ^1^Guangdong Medical University, Dongguan, Guangdong, China; ^2^Clinical Laboratory, Boai Hospital of Zhongshan Affiliated to Southern Medical University, Zhongshan, China; ^3^Reproductive Medical Center, Boai Hospital of Zhongshan Affiliated to Southern Medical University, Zhongshan, China

**Keywords:** COVID-19, immunogenic cell death, biomarker, diagnosis, machine learning

## Abstract

Immunogenic cell death (ICD) serves a critical role in regulating cell death adequate to activate an adaptive immune response, and it is associated with various inflammation-related diseases. However, the specific role of ICD-related genes in COVID-19 remains unclear. We acquired COVID-19-related information from the GEO database and a total of 14 ICD-related differentially expressed genes (DEGs) were identified. These ICD-related DEGs were closely associated with inflammation and immune activity. Afterward, CASP1, CD4, and EIF2AK3 among the 14 DEGs were selected as feature genes based on LASSO, Random Forest, and SVM-RFE algorithms, which had reliable diagnostic abilities. Moreover, functional enrichment analysis indicated that these feature genes may have a potential role in COVID-19 by being involved in the regulation of immune response and metabolism. Further CIBERSORT analysis demonstrated that the variations in the immune microenvironment of COVID-19 patients may be correlated with CASP1, CD4, and EIF2AK3. Additionally, 33 drugs targeting 3 feature genes had been identified, and the ceRNA network demonstrated a complicated regulative association based on these feature genes. Our work identified that CASP1, CD4, and EIF2AK3 were diagnostic genes of COVID-19 and correlated with immune activity. This study presents a reliable diagnostic signature and offers an overview to investigate the mechanism of COVID-19.

## Introduction

Coronavirus infectious disease 2019 (COVID-19), caused by severe acute respiratory syndrome coronavirus 2 (SARS-CoV-2), is a public crisis worldwide since 2019 (Zhong et al., 2020; Kai-Wang To et al., 2021). Due to the mutable nature of SARS-CoV-2, numerous countries are suffering multi-dominant viral infections (Araf et al., 2022; Hadj, 2022). To control its spread, it is crucial to diagnose COVID-19 at an early stage. Accurate molecular diagnostic tests are necessary and valuable to confirm the rapid diagnosis of COVID-19, thus providing valid information for decision-making by the patient, healthcare facilities, and public health organizations (Islam and Iqbal, 2020; Safiabadi Tali et al., 2021; Yuce et al., 2021). Notably, large-scale genome-wide association studies have demonstrated specific disease-related elements in the population (Badua et al., 2021; Robishaw et al., 2021), and there are numerous reports on genes and the prevalence of COVID-19, improving the early diagnosis and clinical management of COVID-19.

Typical pathophysiological mechanisms of COVID-19 include cell death, immune response, oxidative stress, and metabolic activity (Tay et al., 2020; Gattinoni et al., 2021; van Eijk et al., 2021). Cell death and immune infiltration serve a critical role in the formation and progression of COVID-19 (Lee et al., 2020), and immunogenic cell death (ICD) is regarded as a regulative process that could affect cell death and immune infiltration simultaneously (Kroemer et al., 2022). ICD facilitates the activation and recruitment of antigen-presenting cells, thus activating innate and adaptive immune responses (Galluzzi et al., 2020; Minute et al., 2020). Initiating adaptive immunity in ICD is not only enhancing antitumor benefits but is essential for the optimal elimination of infectious etiologies (Galluzzi et al., 2017; Li et al., 2021). Therefore, ICD regulators may have potential diagnostic and therapeutic applications for COVID-19. However, earlier reports focused on only some immune cells and immunological molecules in COVID-19, lacking a perspective on the ICD in COVID-19.

To date, there is growing evidence that multiple cell death modalities, such as ferroptosis, NETosis, and necroptosis, play an important role in the development of COVID-19 (Kang and Wang, 2022; Nishiga et al., 2022; Zhang et al., 2023). Likewise, ICD may also regulate the progression of COVID-19. However, no similar studies have previously explored this specific process, and this study is the first exploration to analyze the relationship between ICD and COVID-19.

In this work, we aimed to determine the vital ICD regulators in the development of COVID-19, thus identifying valuable biomarkers for COVID-19 diagnosis. Subsequently, the correlation between ICD regulators and the infiltrating immune landscape was investigated. Based on the diagnosis biomarkers, the ceRNA network was established, and targeted small molecular agents for COVID-19 were explored. Finally, we validated the identified diagnosis biomarkers with the external dataset. This work offers an in-depth understanding of the developmental mechanisms of COVID-19 at the molecular level and identifies valuable biomarkers.

## Materials and methods

### Data acquisition

The gene expression profiles for COVID-19 and control samples were downloaded from the GEO databases (Barrett et al., 2013). The GSE157103 dataset comprised 100 COVID-19 samples and 26 control samples (Overmyer et al., 2021), and this dataset was deemed to be the training cohort for the principal analysis of this research. The GSE171110 dataset comprising 44 COVID-19 samples and 10 control samples was applied to validate the expression of the feature genes (Levy et al., 2021). Also, the 34 ICD-related genes included in this research were acquired from the previous report (Wang et al., 2021).

### Differential expression analysis

The expression profiles of ICD-related genes in the samples were extracted from the GSE157103 dataset. Next, the limma package was utilized to identify the ICD-related differentially expressed genes (DEGs) between different types of samples (Ritchie et al., 2015). Genes with value of *p* < 0.05 were deemed significant.

### Enrichment of functionality

The expression data were analyzed with functional enrichment to evaluate the potential functionality of promising targets. Gene ontology (GO) is a common approach for identifying the functions of genes, including molecular functions, biological pathways, and cellular components (The Gene Ontology C, 2019; Warwick Vesztrocy and Dessimoz, 2020). Kyoto Encyclopedia of Genes and Genomes (KEGG) pathway enrichment was performed to investigate the genomic information of the DEGs (Kanehisa et al., 2017). Reactome enrichment analysis is also applied to explore gene functions, similar to GO and KEGG analysis (Good et al., 2021). These enrichment approaches were performed with the cluster profiler package (Yu et al., 2012). Additionally, disease ontology was utilized to annotate genes from a disease perspective (Schriml et al., 2022).

### Identification of feature genes

The ICD-related DEGs were further utilized to identify significant feature genes, thus diagnosing COVID-19. The feature identification approach is a procedure of limiting the number of factors, specifically vital for establishing a predictive model (Lee et al., 2021). LASSO regression, Random Forest (RF) algorithm, and SVM-RFE were included in this study to explore feature genes. The “glmnet” package was applied to conduct minimum LASSO regression, thus choosing the linear model and keeping the reliable variables (Engebretsen and Bohlin, 2019). Binomial distribution variables were further presented in the LASSO categorization, with a standard error value as the minimum parameter. Next, according to various dependent decision trees from a training pool, the RF algorithm promotes the precision of the model by randomly limiting the overfitting of individual decision trees (Yang et al., 2020). SVM-RFE can identify the optimal parameters by removing the SVM-derived eigenvectors (Sanz et al., 2018). An SVM module based on the “e1071” package was created to further evaluate the diagnostic value of the selected biomarker in COVID-19 (Jiang et al., 2020). The intersected genes, as the most significant feature genes from these three algorithms, were identified for subsequent analysis. Meanwhile, the predictive reliability of feature genes was evaluated by the receiver operating characteristic (ROC) curve, and the area under the curve (AUC) was further obtained (Park et al., 2004). A logistic regression signature with these feature genes was also established to assess diagnostic ability, and ROC curve was utilized to present this result.

### Pathway correlation analysis

To further investigate the potential pathways of the feature genes, single-gene gene set enrichment analysis (GSEA) and gene set variation analysis (GSVA) were performed with the GSEA package and GSVA package, respectively (Subramanian et al., 2005; Hanzelmann et al., 2013). GSEA was applied to evaluate the distribution landscape of the feature genes of a predetermined collection to explore their attribution to the phenotype. For GSVA, we also applied the KEGG pathway set to conduct enrichment analysis for each feature gene. Next, the limma package was utilized to discuss the difference in the GSVA score of the feature gene’s up- and down-regulated groups (Ritchie et al., 2015).

### Immune cell infiltration analysis

The CIBERSORT algorithm was applied to analyze the normalized gene expression data in the GSE157103 dataset, and the fraction of immune cells was identified (Chen et al., 2018; Overmyer et al., 2021). Violin plots were displayed to present the expressional difference of the immune infiltrating cells. And Spearman correlation analysis was executed to investigate the association between diverse immune infiltrating cells (Fujita et al., 2009). Meanwhile, the correlation between feature genes and immune cells was also explored with a similar method. These results were visualized with the “ggplot2” package (Ito and Murphy, 2013). *p*-value < 0.05 demonstrated statistical significance.

### Drug prediction

Drug Gene Interaction Database (DGIdb^1^) integrates existing literature on drug-gene interactions to provide clinical guidance for personalized treatment of disease. In this report, DGIdb was applied to identify gene-targeted drugs, and DrugBank database was further applied to identify drugs’ structural elements (Wagner et al., 2016; Wishart et al., 2018).

### Establishment of a ceRNA network

Three databases (miRanda, miRDB, TargetScan) were utilized to predict miRNAs (Enright et al., 2003; Agarwal et al., 2015; Chen and Wang, 2020), and the intersections of the predicted miRNAs were identified as the target miRNAs. Afterward, spongeScan was applied to identify the corresponding lncRNAs for miRNAs (Furio-Tari et al., 2016). A ceRNA network using Cytoscape was established predicated on the cross-talk between mRNAs, miRNAs, and lncRNAs (Shannon et al., 2003; Wu et al., 2020).

## Results

### Identification of ICD-related DEGs

**Figure 1:**
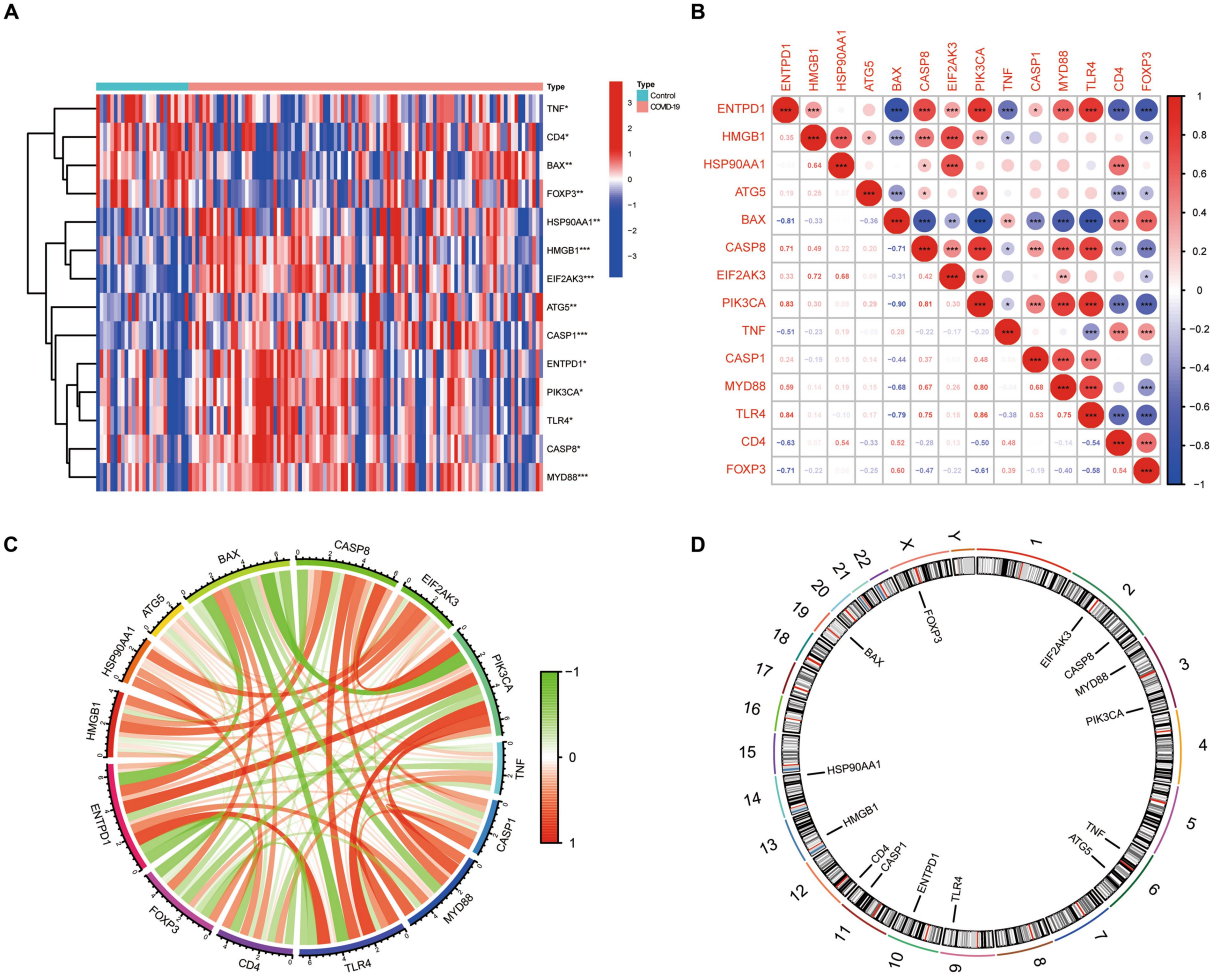
The expression landscape of 14 ICD-related DEGs. **(A)** Heatmap of DEGs between the COVID-19 samples and control samples. **(B,C)** The correlation diagram of DEGs. **(D)** Chromosomal positions of DEGs.

Based on the GSE157103 dataset, 14 ICD-related differentially expressed genes (DEGs) were identified between COVID-19 and control samples. Detailed information on these DEGs were presented in Table 1. Among them, the expression levels of ENTPD1, HMGB1, HSP90AA1, ATG5, CASP8, EIF2AK3, PIK3CA, CASP1, MYD88, and TLR4 were up-regulated, whereas BAX, TNF, CD4, and FOXP3 were down-regulated in COVID-19 samples than that in non-COVID-19 controls (Figure 1A). Next, the correlation between these genes was displayed in Figures 1B,C, and the significant interaction of most genes was observed. Furthermore, Figure 1D also indicated the pattern of the CNV alterations in ICD-related DEGs on their respective chromosomes.

**Table 1:** ICD-related DEGs between COVID-19 samples and control samples.

Gene	*p*-value	Type
ATG5	0.002	Up
BAX	0.003	Down
CASP1	<0.001	Up
CASP8	0.046	Up
CD4	0.011	Down
EIF2AK3	<0.001	Up
ENTPD1	0.028	Up
FOXP3	0.001	Down
HMGB1	<0.001	Up
HSP90AA1	0.004	Up
MYD88	<0.001	Up
PIK3CA	0.023	Up
TLR4	0.038	Up
TNF	0.021	Down

### Enrichment analyses for the ICD-related DEGs

**Figure 2:**
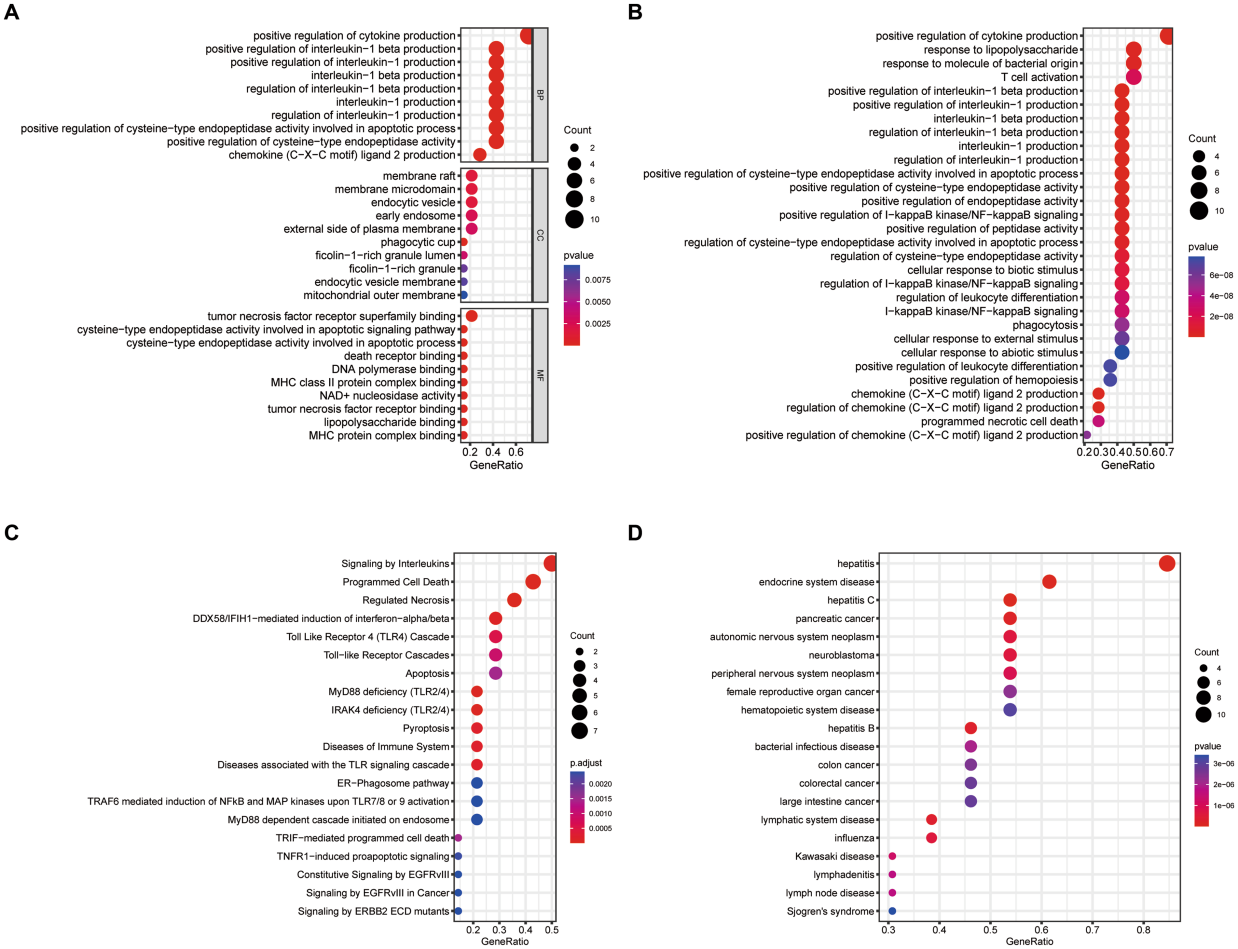
Functional enrichment analysis of ICD-related DEGs. **(A)** GO, **(B)** KEGG, **(C)** Reactome, **(D)** DO enrichment analysis for DEGs.

We conducted GO, KEGG, Reactome, and DO enrichment analysis on ICD-related DEGs, thus discovering the potential molecular biological characteristics of COVID-19. The GO enrichment analyses indicated that these genes were largely involved in the regulation of cytokines (Figure 2A). KEGG pathway analyses showed that the positive regulation of cytokine production, response to lipopolysaccharide, response to molecules of bacterial origin, and T cell activation were enriched (Figure 2B). Moreover, Reactome terms were enriched in signaling by interleukins, programmed cell death, and regulated necrosis (Figure 2C). Interestingly, the ICD-related DEGs were also markedly enriched in inflammation and tumor-related signatures (Figure 2D). These findings demonstrated that ICD-related DEGs may serve a critical role in the etiology of COVID-19 by engaging in the regulation of cytokines and diverse cell death processes.

### Identification of ICD-related diagnostic genes for COVID-19

**Figure 3:**
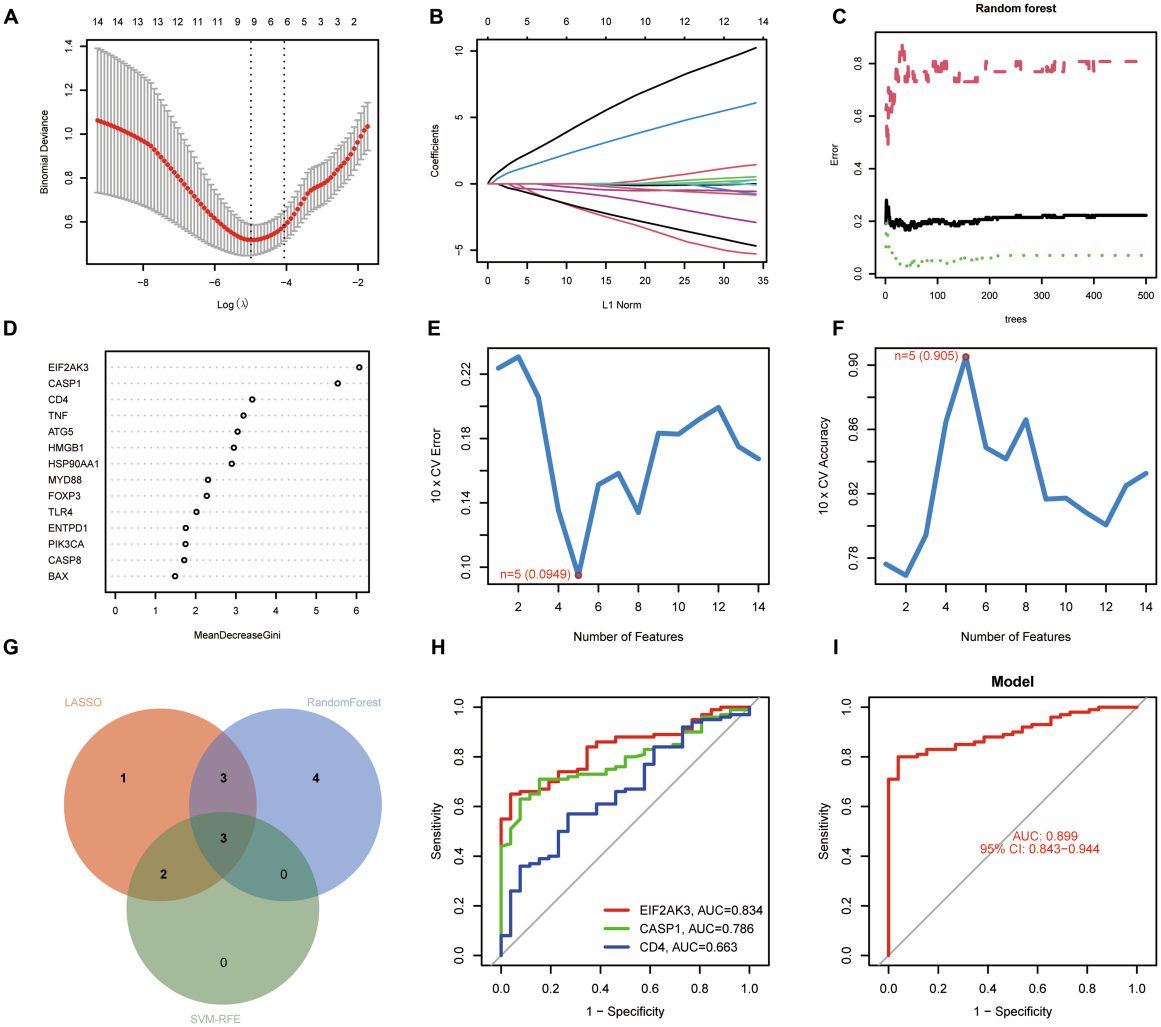
Identification of diagnostic genes for COVID-19. **(A,B)** Establishment of the LASSO model. **(C,D)** Screening biomarkers based on random forest (RF) algorithm. **(E,F)** Results of screening biomarkers based on SVM-RFE algorithm. **(G)** Venn diagram showing the intersected genes. **(H)** ROC curves for the feature genes. **(I)** Logistic regression model to identify the AUC of disease samples.

For exploring the potential pathogenesis of COVID-19, we further evaluated the diagnostic values of ICD-related DEGs. Machine learning algorithms (LASSO, RF, and SVM-RFE) were selected and executed to identify the significant ICD-related DEGs, thus distinguishing COVID-19 from control samples. LASSO regression analysis was performed to select nine feature genes with statistically significant univariate parameters (Figures 3A,B). RF incorporating feature selection was utilized to investigate the relationship between error rate, number of classification trees, and 14 genes with relative importance (Figure 3C). Meanwhile, the respective importance of these genes was also presented in Figure 3D. Next, SVM-RFE analysis indicated that the SVM model based on five feature genes had an optimum generalization performance (Figures 3E,F). The feature genes from the abovementioned algorithms were intersected, and three feature genes (CASP1, CD4, and EIF2AK3) were determined for subsequent analysis (Figure 3G). To reveal the diagnostic accuracy of feature genes in distinguishing COVID-19 from non-COVID-19 controls, ROC curves were plotted for the three feature genes. As depicted in Figure 3H, the AUCs for the three feature genes were higher than 0.6, indicating that these genes have good diagnostic performance. Furthermore, we established a logistic regression model based on the three feature genes, and the ROC curves suggested that the logistic regression model differentiated COVID-19 and control samples with AUC = 0.899 (Figure 3I). This evidence demonstrated that the three feature genes can be considered valuable diagnostic genes for COVID-19, and the diagnostic model incorporating the three feature genes may have greater accuracy than individual feature genes.

### GSEA and GSVA analysis

**Figure 4:**
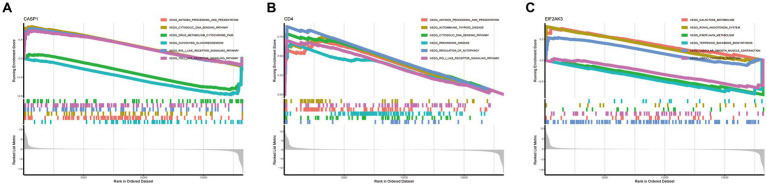
Single-gene GSEA-KEGG pathway analysis in CASP1 **(A)**, CD4 **(B)**, and EIF2AK3 **(C)**.

**Figure 5:**
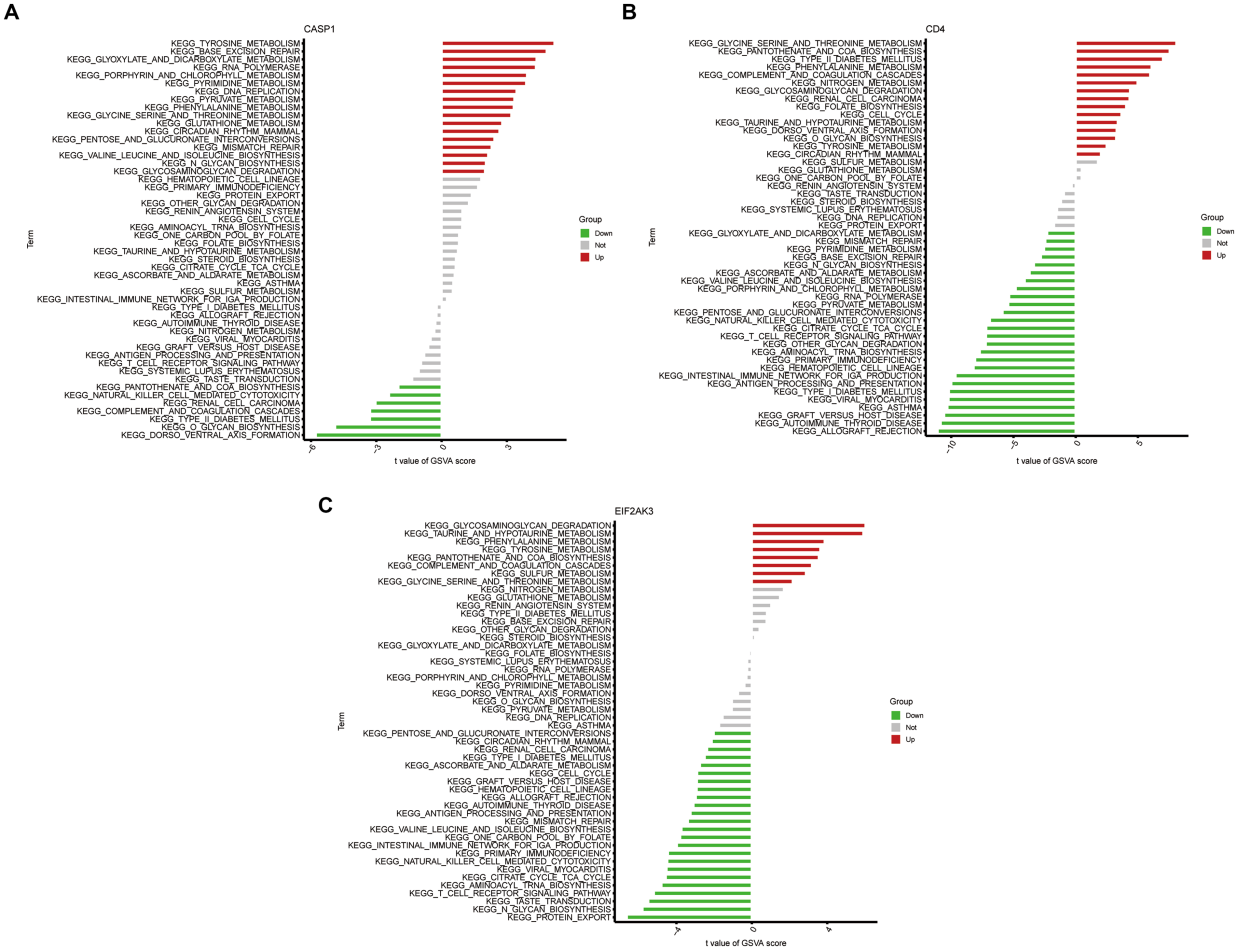
High- and low-expression groups based on the expression levels of each feature gene combined with GSVA in CASP1 **(A)**, CD4 **(B)**, and EIF2AK3 **(C)**.

To further determine the underlying role of feature genes to identify characteristic differences between COVID-19 samples and control samples, a single-gene GSEA-KEGG pathway analysis was performed. The top six pathways enriched for individual feature genes were presented in Figures 4A–C. After a systematic analysis, we observed that CASP1 and CD4 were activated in immune response (such as antigen processing and presentation) and RIG-I-like receptor signaling pathway. Meanwhile, EIF2AK3 was closely associated with the metabolism-related activity. Afterward, we found the differentially enriched GSVA pathways between the high- and low-expression groups of each feature gene (Figure 5). The findings indicated that the high expression of CASP1 in COVID-19 may induce this disease by “tyrosine metabolism,” “base excision repair,” and “glyoxylate and dicarboxylate metabolism,” while down-regulation of CASP1 promoted “DORSO_VENTRAL_AXIS_FORMATION,” “O_GLYCAN_BIOSYNTHESIS,” and “VALINE_LEUCINE_AND_ISOLEEUCINE_BIOSYNTHESIS.” CD4, whose expression was limited in COVID-19 samples, was significantly correlated with the immune response (“AUTOIMMUNE THYROID DISEASE,” “GRAFT VERSUS HOST DISEASE,” and “ALLOGRAFT REJECTION”). Moreover, the high expression of CD4 activated the pathways such as “GLYCINE SERINE AND THREONINE METABOLISM,” and “PANTOTHENATE AND COA BIOSYNTHESIS.” Similarly, in the EIF2AK3 over-expression group, “GLYCOSAMINOGLYCAN DEGRADATION,” and metabolism-related pathways (“TAURINE AND HYPOTAURINE METABOLISM,” “PHENYLALANINE METABOLISM,” “TYROSINE METABOLISM”) were enriched. The low-expressed EIF2AK3 served a critical function in the protein export, and biosynthesis process (such as N-GLYCAN biosynthesis and AMINOACYL TRNA biosynthesis).

### Immunological infiltration analysis

**Figure 6:**
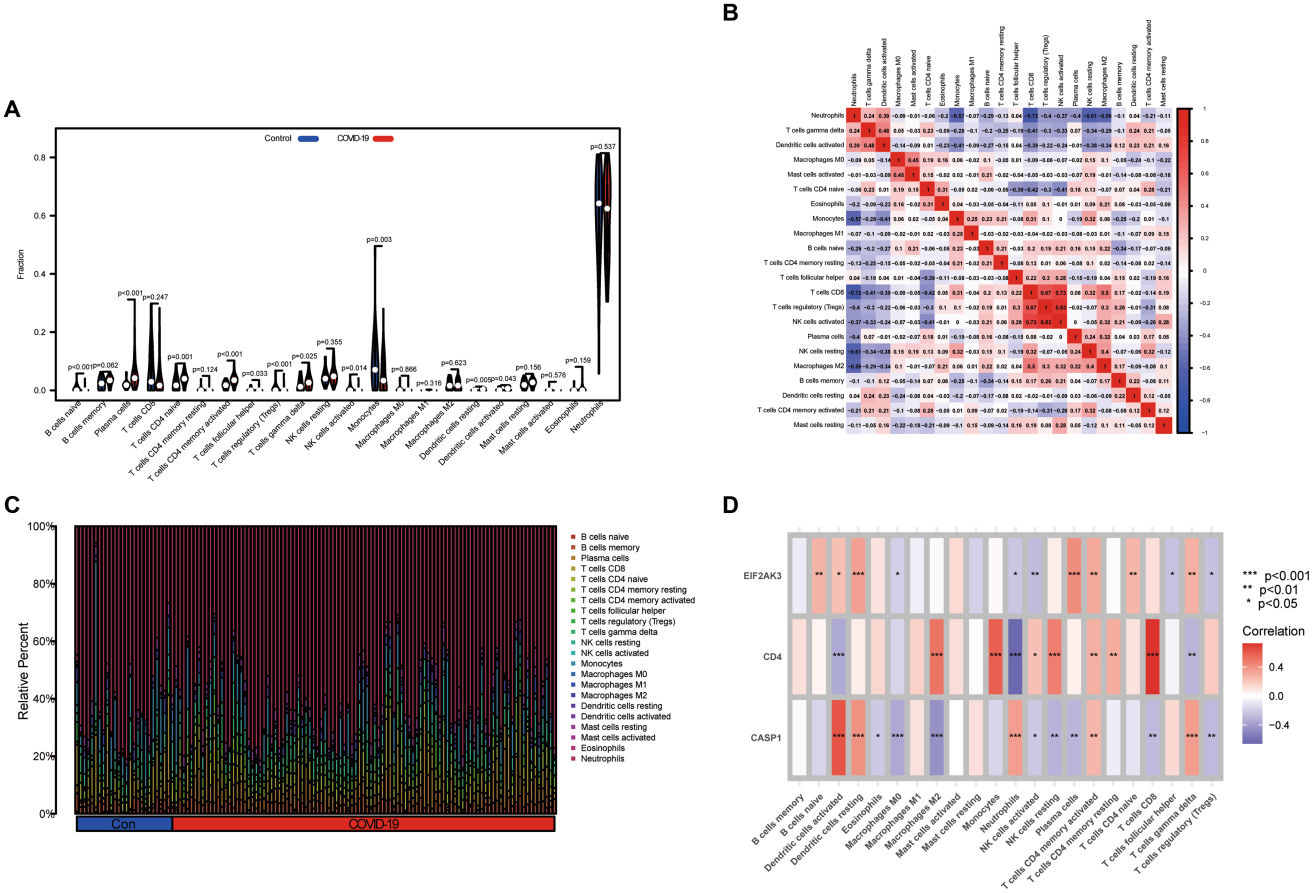
Immune landscape analysis. **(A)** The differences in immune cells from the immune microenvironment between COVID-19 patients and normal samples. **(B)** The correlation diagram of immune cells. **(C)** The bar plot presenting the proportion of infiltrated immune cells calculated by the CIBERSORT algorithm. **(D)** The correlation between feature genes and immune cells.

Based on the CIBERSORT algorithm, the infiltration levels of 22 types of immune cells between COVID-19 and control samples were evaluated. As presented in Figure 6A, compared with the control samples, naïve B cells, follicular helper T cells, Tregs, activated NK cells, monocytes, and activated mast cells were less enriched, while plasma cells, CD4 naïve T cells, activated CD4 memory T cells, γδ T cells, resting dendritic cells, and activated dendritic cells were more enriched in COVID-19 sample. Subsequently, after performing a correlation analysis of infiltrating immune cells, we identified diverse pairs of interacting immune cells (Figure 6B). Similarly, the proportions of different immune cells between the COVID-19 and control samples had a significant difference (Figure 6C). Furthermore, we also explored the relevance of three diagnostic genes to immune cells. As shown in Figure 6D, Pearson correlation analysis suggested that activated dendritic cells, neutrophils, activated NK cells, activated CD4 memory T cells and γδ T cells were all associated with three feature genes (CASP1, CD4, and EIF2AK3). These results suggested that alterations in the immune microenvironment of COVID-19 samples correlated with these three genes.

### Prediction of feature gene-targeted drugs

**Figure 7:**
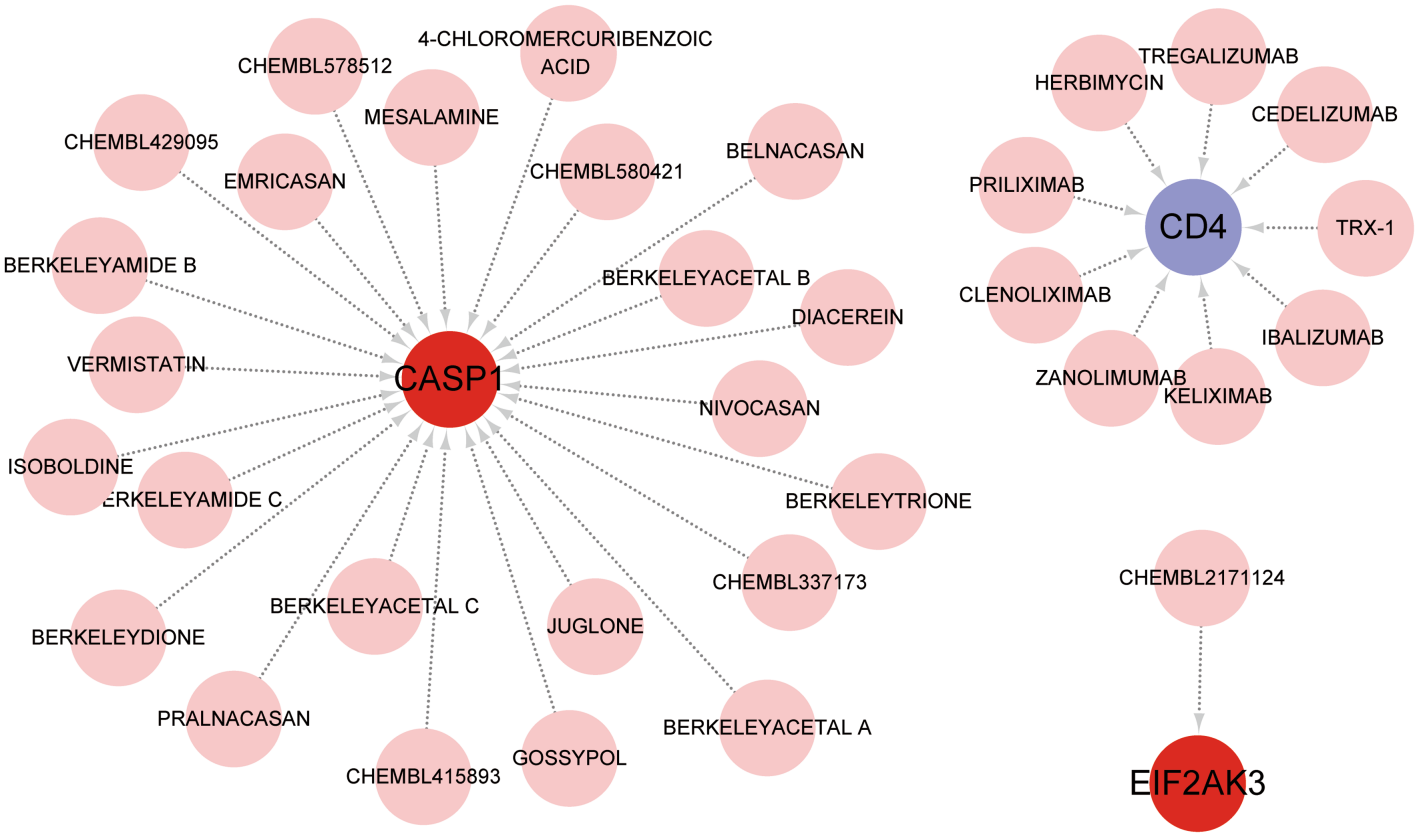
Prediction of feature gene-targeted drugs based on DGIdb database.

DGIdb was utilized to identify the underlying drug or molecular compounds that could regulate the expression of feature genes in the setting of COVID-19. As demonstrated in the drug-gene interaction network (Figure 7), 33 drugs or molecular compounds targeting feature genes were identified, including 23 for CASP1, 9 for CD4, and 1 for EIF2AK3. The results suggest that CASP1 is more likely to be a valuable target for drug development against COVID-19. Interestingly, the drugs that interacted with different genes were not observed.

### A ceRNA network based on diagnostic genes

**Figure 8:**
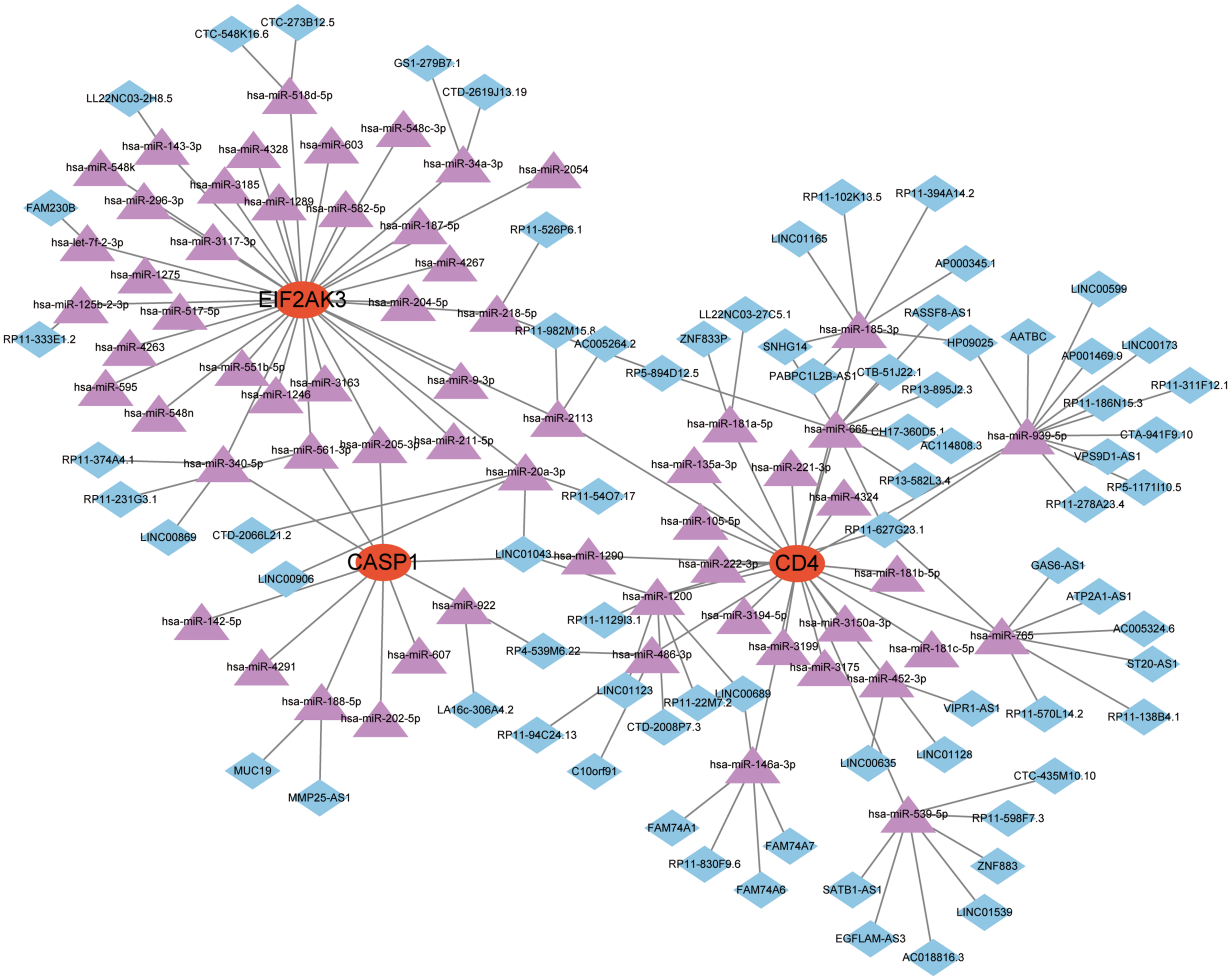
A ceRNA network based on three feature genes.

Recently, the ceRNA network is emerging as a hot topic and serves a pivotal role in controlling gene activity. The ceRNA network was established with miRNA as a bridge to determine the relationship between target gene, mRNA and lncRNA by interacting miRNA response factors. Referring to epigenetic regulators, miRNAs are considered valuable therapeutic options for diverse diseases. The miRNAs associated with 3 feature genes were explored from three online databases, and the interaction demonstrated by these databases were also identified. A total of 63 miRNAs and 3 mRNAs formed 67 interaction pairs. Additionally, we investigated the lncRNAs that corresponded to regulator miRNAs based on the spongeScan database and further established a ceRNA network of lncRNA-miRNA-mRNA. Finally, this network was comprised of 145 nodes (3 genes, 63 miRNAs, and 75 lncRNAs) and 151 edges (Figure 8).

### Expression of diagnostic genes in the validation cohort

**Figure 9:**
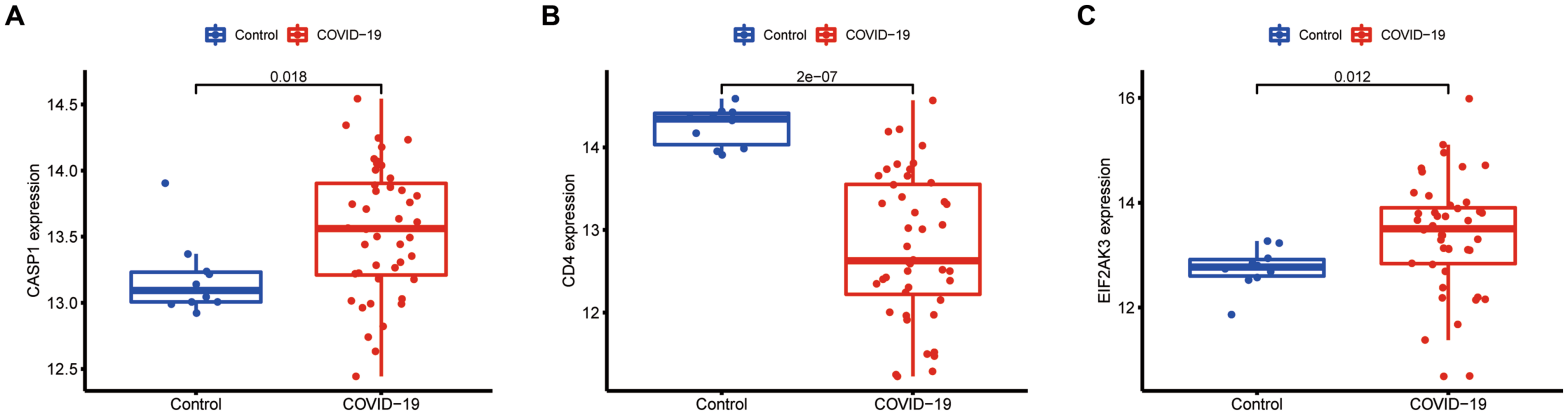
Expression difference of the feature genes (**A**, CASP1; **B**, CD4; **C**, EIF2AK3) between COVID-19 and normal samples in the validation cohort (GSE171110 dataset).

Additionally, the expression of three diagnostic genes (CASP1, CD4, and EIF2AK3) was also explored in the GSE171110 dataset. We observed that the expression levels of these diagnostic genes were consistent with the GSE157103 dataset (Figure 9). Among them, the expression of CD4 (*p* < 0.001) in COVID-19 samples was lower than that of control samples, while CASP1 (*p* = 0.018) and EIF2AK3 (*p* = 0.012) were higher in COVID-19 samples.

## Discussion

COVID-19 has been ravaging the world for years, and its specific cause remains unclear. The mutational and variable nature of COVID-19 renders it impossible to explain it with changes in a single gene. Hence, the management of COVID-19 patients cannot be implemented by a method, instead diverse methods, including multi-targeted agents and multi-modalities treatment (Fahmi et al., 2021; Rantam et al., 2021). It is essential to explore novel and promising biomarkers for the early diagnosis and management of COVID-19 (Zhong et al., 2020; Gattinoni et al., 2021). Notably, virous cell death forms have been observed to be significantly correlated with the progression of COVID-19, but the association between COVID-19 and ICD has not yet been clarified. Herein, we attempted to identify diagnostic biomarkers pertaining to COVID-19 and delve into the role exerted by ICD within COVID-19.

In this work, we identified 14 ICD-related DEGs between COVID-19 and normal samples, including 10 up-regulated and 4 down-regulated genes. Afterward, functional enrichment analysis of these DEGs was executed, and these genes presented close associations to the regulation of cytokines and cell death signals (such as regulation of cysteine-type endopeptidase activity involved in the apoptotic process, programmed cell death, and regulated necrosis). According to these findings, COVID-19 displayed immunological processes and cell death involvement, thus inducing inflammation and infection.

We applied three different machine learning algorithms (LASSO, RF, and SVM-RFE), each with its advantages. LASSO regression identifies parameters by finding the variable with the lowest incidence of categorical error (Tibshirani, 1997). RF algorithm is an integrative algorithm consisting of decision trees, which is applied to train and predict samples (Simsekler et al., 2021). SVM-RFE has acquired extensive attention in ranking traits and selecting important traits for categorization (Sanz et al., 2018). Finally, CASP1, CD4, and EIF2AK3 were identified and were valuable for further explorations, which demonstrated that our prediction displayed reliability with the integrated approaches.

CASP1, a member of the caspase family, serves a critical role in the execution process of cell apoptosis (Kapplusch et al., 2019). The expression of CASP1 was regulated, and the inflammasome activated by CASP1-NLRP3 dependent pathway can subsequently secrete IL-1β and IL-18, thus leading to dysfunctional autophagy (Wang et al., 2017). Upregulation of CASP1 in COVID-19 was reported in a prior report, consistent with our findings (Yang et al., 2022). CD4 encodes the CD4 membrane glycoprotein of T lymphocytes, and CD4+ T cell plays a critical role in anti-viral immunity (Matthias et al., 2002; Sharma et al., 2005). CD4 mediates various biological processes of immune cells. Meanwhile, immune cells, primarily comprising T cells, B cells, and macrophages, crucially affect the pathogenesis of COVID-19 (Tay et al., 2020). EIF2AK3, as a metabolic stress-sensing protein kinase, inhibits protein translation and regulated pro-survival autophagy (You et al., 2021). These genes displayed significantly different expressions between COVID-19 and normal samples, and this result was also observed in the validation cohort. Furthermore, GSEA and GSVA for three feature genes were performed, and we observed that these genes were significantly associated with immune response and metabolism-related activities. Based on the abovementioned findings, CASP1, CD4, and EIF2AK3 have the potential to influence the progression of COVID-19 and serve as diagnostic biomarkers, whereas this finding remains to be confirmed by further clinical trials.

To more comprehensively investigate the effects played by the abundance of immune cells in COVID-19, this research conducted CIBERSORT for evaluating the infiltrating immune status within COVID-19. The abundance of naïve B cells, follicular helper T cells, Tregs, activated NK cells, monocytes, and activated mast cells decreased, while the abundance of plasma cells, CD4 naïve T cells, activated CD4 memory T cells, γδ T cells, resting dendritic cells, and activated dendritic cells increased, potentially demonstrating the relevance of COVID-19 initiation and development. It is common knowledge that these cells are an essential part of human adaptive immunity, and these cells may be potential factors for COVID-19 pathogenesis (Sette and Crotty, 2021). Meanwhile, three feature genes presented a significant correlation with diverse immune cells, which also confirmed the autoimmunogenicity of these genes. Although this is widely recognized, the molecular mechanisms and functions of immune cell infiltration in COVID-19 need to be further investigated urgently.

Finally, the feature gene for gene-targeted drugs and the ceRNA network were explored. A diagram of 33 drugs with promising therapeutic efficacy against COVID-19 was displayed. The performance of the selected drugs was validated for diverse diseases; however, the mechanisms of many drugs were unclear. Further molecular experiments and clinical investigations are necessary to identify the drugs that are valuable for COVID-19 treatment. The target miRNAs and the target lncRNAs were predicted for CASP1, CD4, and EIF2AK3, and a ceRNA network was established with Cytoscape. This network proposes a potential mechanism for selected genes to be regulated at the transcriptome level.

Inevitably, these are some restrictions of this study. Firstly, this study was performed on public databases, and additional laboratory experiments are necessary. Secondly, the association between these results in this study and clinical variables was worthy of future analysis and validation. However, this study could still reflect convincing evidence to further investigate the significance of ICD-related genes in the treatment and diagnosis of COVID-19.

## Conclusion

Taken together, we presented that differentially expressed genes: CASP1, CD4, and EIF2AK3 might be considered as the diagnostic biomarker for COVID-19 and characteristic immunological infiltration could influence the systemic immune microenvironment in the pathogenesis of COVID-19. This work may reveal potential biomarkers related to immune regulation that ultimately led to COVID-19 infection. Additional leading-edge approaches, such as single-cell sequencing method, will further offer promising perspectives into COVID-19 development and drug targets.

## Data availability statement

The original contributions presented in the study are included in the article/supplementary material, further inquiries can be directed to the corresponding author.

## Ethics statement

Ethical review and approval was not required for the study on human participants in accordance with the local legislation and institutional requirements. Written informed consent for participation was not required for this study in accordance with the national legislation and the institutional requirements.

## Author contributions

JZ and CY: conceptualization. JZ and KW: methodology. ZS: software and data curation. JZ, KW, and ZS: validation. CY: formal analysis, writing—review and editing, and project administration. JZ: investigation, writing original draft preparation, visualization, and supervision. KW: resources. All authors contributed to the article and approved the submitted version.

## Conflict of interest

The authors declare that the research was conducted in the absence of any commercial or financial relationships that could be construed as a potential conflict of interest.

## Publisher’s note

All claims expressed in this article are solely those of the authors and do not necessarily represent those of their affiliated organizations, or those of the publisher, the editors and the reviewers. Any product that may be evaluated in this article, or claim that may be made by its manufacturer, is not guaranteed or endorsed by the publisher.

## Footnotes

^1^ https://dgidb.org/
